# Process monitoring in intensive care with the use of cumulative expected minus observed mortality and risk-adjusted *p *charts

**DOI:** 10.1186/cc3996

**Published:** 2006-02-14

**Authors:** Jerome GL Cockings, David A Cook, Rehana K Iqbal

**Affiliations:** 1 Department of Intensive Care Medicine, Royal Berkshire Hospital, Reading, Berkshire RG1 5AN, UK; 2 Intensive Care Unit, Princess Alexandra Hospital, Brisbane, Queensland, Australia Ipswich Road, Wooloongabba, Brisbane QLD, 4000, Australia; 3 Department of Intensive Care Medicine, Royal Berkshire Hospital, Reading, Berkshire RG1 5AN, UK

## Abstract

**Introduction:**

A health care system is a complex adaptive system. The effect of a single intervention, incorporated into a complex clinical environment, may be different from that expected. A national database such as the Intensive Care National Audit & Research Centre (ICNARC) Case Mix Programme in the UK represents a centralised monitoring, surveillance and reporting system for retrospective quality and comparative audit. This can be supplemented with real-time process monitoring at a local level for continuous process improvement, allowing early detection of the impact of both unplanned and deliberately imposed changes in the clinical environment.

**Methods:**

Demographic and UK Acute Physiology and Chronic Health Evaluation II (APACHE II) data were prospectively collected on all patients admitted to a UK regional hospital between 1 January 2003 and 30 June 2004 in accordance with the ICNARC Case Mix Programme. We present a cumulative expected minus observed (E-O) plot and the risk-adjusted *p *chart as methods of continuous process monitoring. We describe the construction and interpretation of these charts and show how they can be used to detect planned or unplanned organisational process changes affecting mortality outcomes.

**Results:**

Five hundred and eighty-nine adult patients were included. The overall death rate was 0.78 of predicted. Calibration showed excess survival in ranges above 30% risk of death. The E-O plot confirmed a survival above that predicted. Small transient variations were seen in the slope that could represent random effects, or real but transient changes in the quality of care. The risk-adjusted *p *chart showed several observations below the 2 SD control limits of the expected mortality rate. These plots provide rapid analysis of risk-adjusted performance suitable for local application and interpretation. The E-O chart provided rapid easily visible feedback of changes in risk-adjusted mortality, while the risk-adjusted *p *chart allowed statistical evaluation.

**Conclusion:**

Local analysis of risk-adjusted mortality data with an E-O plot and a risk-adjusted *p *chart is feasible and allows the rapid detection of changes in risk-adjusted outcome of intensive care patients. This complements the centralised national database, which is more archival and comparative in nature.

## Introduction

A contemporary model of a health care system is that of a complex adaptive system [1] with multiple nested interconnected parts that evolve, interact and adapt over time. During an episode of care, the quality of care delivered by the system results from an interaction between the patient and all interrelated parts of the system. All changes made within the system will affect all patients, to a greater or lesser extent. Isolated analyses may not be informative, as changes planned for beneficial, direct consequences may trigger indirect, adaptive effects that can be detrimental overall. Constraints of rationing and redistribution of scarce resources, the paucity of rigorous examination of critical care practice and the adaptive and emergent features of a complex interactive system undermines the logic of expecting the application of pockets of experimental evidence to lead naturally to improved outcomes for all patients. It is therefore not enough to incorporate the best empirical practice conscientiously into each step in the patient encounter. It is important that evidence-based practice must incorporate evidence of benefit in the context of the particular health care environment of interest, and that a global measure of efficacy be employed.

It is difficult to measure the quality of an intensive care service. Death statistics are potentially misleading and are not indicative of just the quality of the system: there are influences of patient numbers, severity of illness and diagnosis. It is desirable to control for confounding factors, and several domains have consistent and reproducible associations with risk of death [2]. In critical illness, these domains are the patient's severity of acute disturbance (captured by physiological observations and laboratory investigations), physiological reserve (captured by age and co-morbidities), the diagnosis or procedure, and also less influential variables such as lead-time, emergency status and referral source. This relationship is not purely deterministic because random effects and unmeasured factors, such as the effect of the quality of the process of care, contribute to outcome for an individual patient [3].

A validated model that accurately estimates the probability of patient death such as the UK Acute Physiology and Chronic Health Evaluation II (APACHE) II system can be used to control for severity of illness and case mix [4,5]. Such systems will be familiar to critical care clinicians. Potentially, the effects on mortality of both random effects and unmeasured factors (such as the quality of care) can be teased out. By continuous real-time comparison of the predicted and observed outcomes, the process of care can be monitored with regard to whether the risk-adjusted mortality equals, exceeds or falls below the expectation of the model. The validated external model is analogous to 'in-control' specifications of an industrial process. In the UK a centralised national database, the Intensive Care National Audit & Research Centre (ICNARC) Case Mix Programme (CMP), operates from a central hub that issues reports based on pooled and collected data. There are delays inherent in data collation from multiple other sites, and centrally generated reports can return months after the collection period, making them of archival, rather than formative, value.

Grigg and Farewell [6] have reviewed risk-adjusted charts. Plots of the cumulative difference between expected and observed outcomes (E-O plots) provide a qualitative and intuitive representation of accumulating patient data, and methods of incorporating control limits have been described [7,8]. Risk-adjusted *p *charts lack the power to detect small changes in performance, do not accumulate evidence over time, are vulnerable to the effects of multiple testing, and have an obligate delay to finalise a sample period (that is, a month of data) before an alert can be recognised, irrespective of the magnitude of the difference between observed and predicted outcomes. However, risk-adjusted *p *charts complement the expected minus observed (E-O) plot and are simple to construct and explain. With relatively common event rates and adequate patient numbers, they may have a performance that approaches the risk-adjusted sequential techniques. Risk-adjusted CUSUM (cumulative sum) charts, such as the charts by Steiner and colleagues. [9] and the resetting probability ratio test charts [10] and the Sets method [11], can be more sensitive for detecting differences in performance. Arguably, these can be more difficult to design for the optimal detection of changes with an acceptable false alarm rate, and they can be difficult to explain to managers, clinicians and non-statisticians.

The purpose of this paper is to evaluate a simple method of local outcome analysis to supplement the ongoing central reporting system. We have selected the E-O chart and the risk-adjusted *p *chart mortality as techniques that are easy to apply and that track differences between predicted and observed outcomes. These combine a rapidly responsive, qualitative evaluation with a robust statistical evaluation. We use these alongside the familiar standardised mortality ratio (SMR) chart and comment on how this local approach complements the central collation and reporting paradigm of outcome monitoring from a national, centralised database.

## Materials and methods

All patient episodes at a regional intensive care unit (Royal Berkshire Hospital (RBH), Reading, UK) from 1 January 2003 and 30 June 2004 were studied under local ethics committee approval. Data were collected prospectively in accordance with the ICNARC CMP [4,5,12-14]. Clinical, demographical and physiological data were collected on admission and during the first 24 hours in intensive care. The probability of mortality was calculated with the APACHE II system [15] but using a model optimised for the UK population, the UK APACHE II [4,13,14]. Data were collected in accordance with the ICNARC CMP, a national comparative audit of intensive care outcome. More details of the CMP have been described elsewhere [5,12] and can be found on the ICNARC website [16]. The endpoint was survival status at discharge from RBH. In accordance with the national ICNARC CMP dataset (ICMPDS version 2) [5], episodes were excluded for patients less than 16 years old, for intensive care unit (ICU) admissions lasting less than eight hours, admissions for primary burns, admissions after coronary artery bypass grafting, transfers in from another ICU, readmissions within the same hospital stay or admissions lacking all 12 physiological variables. Data were collected with a Clinical Information System (Eclipsys Technologies Corporation, Boca Raton, FL, USA). The ICNARC data subset was then extracted from this with a specially developed database program (Wardwatcher; Critical Audit, London, UK). All data were verified by a trained data collection nurse and diagnoses were checked by two doctors, one of whom was an intensive care physician. A random sample of 5% of patients' physiological and clinical data were extracted and independently verified. All patients were followed up until death or hospital discharge.

Model fit was assessed with a calibration curve, and model discrimination was measured by the area under the receiver operating characteristic curve, approximated by the trapezoidal method and estimation of 95% confidence intervals [17,18].

The cumulative E-O mortality chart uses patients indexed by order of admission to the ICU. A mathematical description is provided in Additional file 1. It has been described previously as a variable life adjusted display (VLAD) [19] and a cumulative risk adjusted mortality (CRAM) chart [7]. For each patient the probability of in-hospital death was estimated, and in-hospital outcome (0 for a hospital survivor, 1 for an in-hospital death) was recorded. The estimates of probability of death minus the observed outcomes were then accumulated for sequential admissions. The cumulative difference between the expected and observed number of deaths is displayed on the *y*-axis, for the sequence of patients. The *x*-axis displays sequential patient admissions, although the date of ICU admission is used on the label for ease of interpretation.

**Figure 1:**
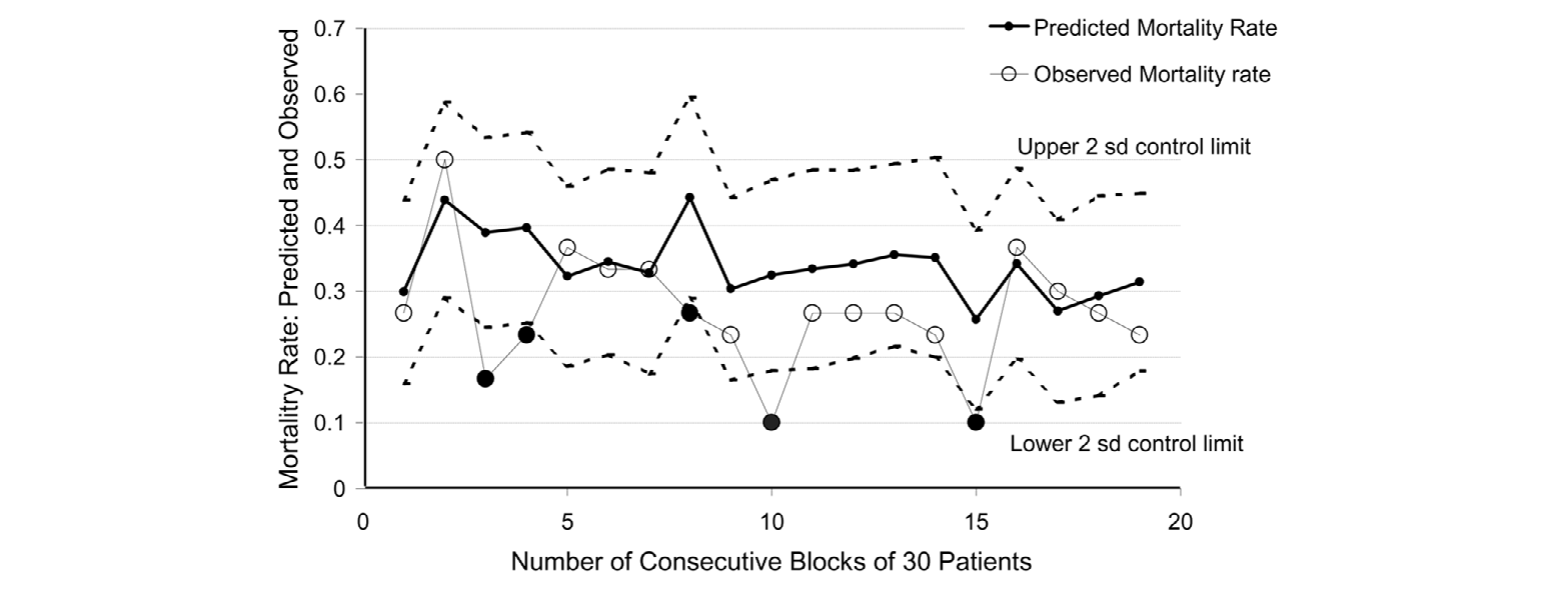
Risk-adjusted *p *chart by blocks of 30 patients. Probability of death estimated with UK APACHE, Royal Berkshire Hospital, 1 January 2003 to 30 June 2004.

**Figure 2:**
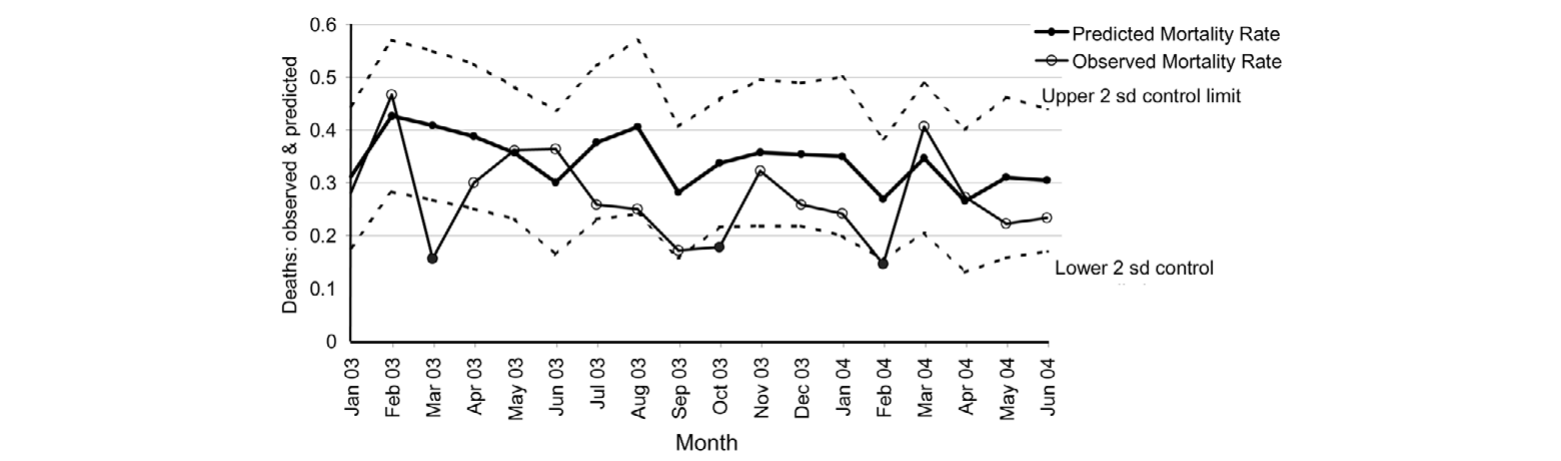
Risk-adjusted *p *Chart by month. Probability of death estimated with UK APACHE, Royal Berkshire Hospital, 1 January 2003 to 30 June 2004.

The risk-adjusted *p *chart [20] is a control chart plotting the observed mortality rate and expected mortality rate in groups of patients. It is presented in detail in Additional file 1. In this case we have chosen 2 units of the estimated SD above and below the expected mortality rate as the upper and lower control limits. A single, independent, observation outside the control limits will occur by chance about 5% of the time. Figure 1 shows the risk-adjusted *p *chart of blocks of 30 consecutive patients. Figure 2 presents the same data but with the patients grouped into monthly blocks of variable sizes, as caseload varies from month to month.

SMRs were calculated with 95% confidence intervals [21] from samples of three months of cases, using observed mortality rate divided by the mean expected risk of death.

## Results

Patients excluded from scoring in accordance with the UK APACHE II system rules are given in Table 1, comparing RBH ICU and the ICNARC CMP data for 2003. Characteristics of the patient sample are given in Table 2, with the ICNARC data for 2003 for comparison. The RBH mean APACHE II score was 20.8; the observed hospital mortality rate during the study period was 28.9% overall, and 26% for those included for severity scoring. The predicted mortality rate was 33.5% (SMR 0.78).

**Figure 3:**
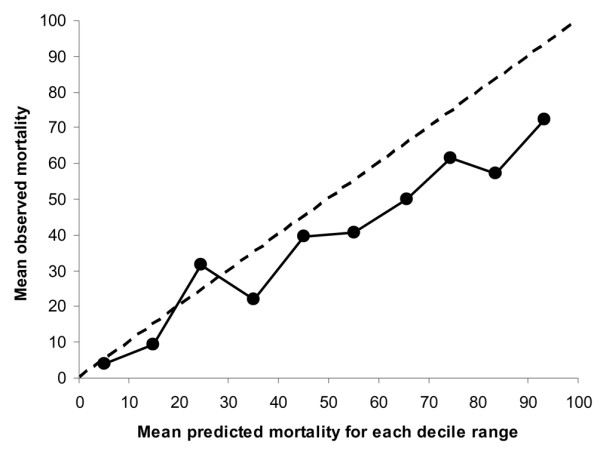
Calibration curve of the UK APACHE II Model at the Royal Berkshire Hospital, 1 January 2003 to 30 June 2004.

**Figure 4:**
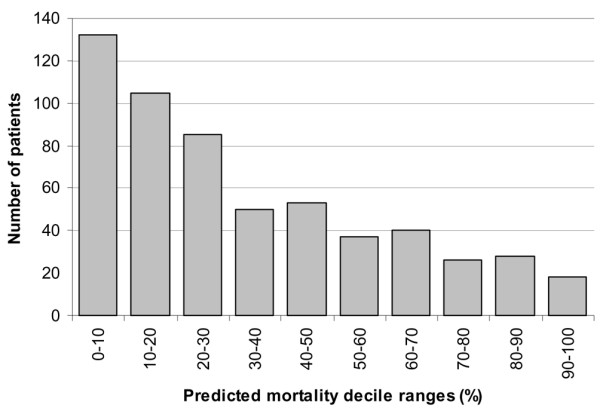
Histogram of patients in risk of death ranges: UK APACHE, Royal Berkshire Hospital, 1 January 2003 to 30 June 2004.

The calibration curve is displayed in Figure 3, showing an overestimate of risk of death in patient risk ranges above 30%. A histogram of patient numbers in each of the risk of death ranges (Figure 4) shows that most of the patients were in the lower ranges, below 30% predicted mortality. The area under the receiver operating characteristic curve for our data is 0.78 (95% confidence interval 0.74 to 0.82). Although the case mix is similar to that of the ICNARC dataset, the UK APACHE II model overestimates patient risk of death for the RBH patient population, notably in patients with a higher risk of death.

**Figure 5:**
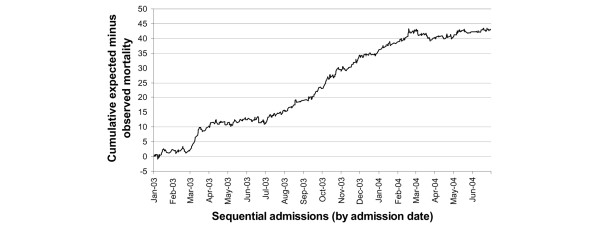
Cumulative expected minus observed mortality chart. Probability of death estimated with UK APACHE, Royal Berkshire Hospital, 1 January 2003 to 30 June 2004.

Figure 5 is the cumulative E-O plot for the series of admissions. Generally, there is a positive gradient, supporting the observation that the UK APACHE II predictions consistently overestimate the risk of death, although some variations in the slope are observed. These variations represent either random fluctuations in the charting process or real but transient changes in the quality of care.

The risk-adjusted *p *charts (Figures 1 and 2) show that for some periods the observed mortality rate was below the lower 2 SD control limit. The mortality rates observed in the blocks of 30 patients numbered 3, 4, 8, 10 and 15 were all below the lower control limits. Figure 2, presenting monthly data, shows that in March 2003, October 2003 and February 2004 the observed mortality rate was below the 2 SD control limits. Even accounting for multiple testing this is very likely to represent a patient mortality rate below that predicted.

**Figure 6:**
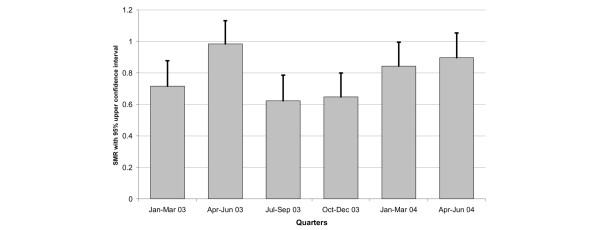
Standardised mortality ratios by quarter, with 95% confidence intervals. Probability of death estimated with UK APACHE, Royal Berkshire Hospital, 1 January 2003 to 30 June 2004

Figure 6 shows SMRs for each quarter, with 95% confidence intervals. In all quarters, the value of the SMR fell below 1, and in three of the six quarters the upper 95% confidence interval did not extend to 1.

## Discussion

This report presents an example of a monitoring paradigm in which local performance is compared with an ICU cohort with the use of a validated risk adjustment model. The UK APACHE II model has been validated across the UK population [13,14,22]. This represents an external performance benchmark and is analogous to an 'in-control' performance specification.

The number of deaths observed was less than that predicted by the UK APACHE II model. Nationally, in ICUs participating in the ICNARC CMP, the UK APACHE II model underestimates mortality (SMR 1.11 for 2003), while overestimating it in our institution over a similar period and with a similar case mix (SMR 0.78) (Tables 1 and 2). Differences in ICU model performance between sites have been attributed to imperfect model generalisation and to differences in model performance arising from different interpretations of the model rules, varying data collection methods [23,24], variations in case mix [25-29] and organisational factors [30]. The overall difference between the predicted and observed mortality is likely to be due to a combination of several factors.

Risk-adjusted control charts are not new to health care, but they are not used widely in intensive care medicine. Lovegrove and colleagues. [19] and Poloniecki and colleagues. [7] described the monitoring of outcome from cardiac surgery with the use of risk-adjusted control charting, and subsequent publications have provided further examples in cardiac surgery [9,10,31-33], heart and lung transplantation [34] and myocardial infarction [8,35].

In the critical care literature there have been few examples of control charts. Chamberlin and colleagues. [36] reported tracking the severity of illness rather than the outcomes of ICU care. Cook and colleagues. [20] described the risk-adjusted *p *charts and an application of the risk-adjusted CUSUM in an Australian ICU, using the APACHE III model as a risk adjustment tool. Improvement in performance was temporally related to increased senior staffing levels and enhanced ongoing interdisciplinary review of practice, quality improvement and educational activities.

Risk-adjusted control charts can track differences between expected and observed performance. In this illustration, the UK APACHE II model overpredicted the risk of death to some extent during the 18-month period of analysis. This is apparent from the upward slope on the qualitative E-O chart. The risk-adjusted *p *chart strongly suggests that the observed mortality rates of blocks of 30 patients and the mortality rates for months of variable case load were often significantly less than the predicted mortality rate. This observation is supported by the conventional quarterly SMR analysis.

Local prospective monitoring has recently been advocated in medicine [37]. It is a fundamentally different view of quality measurement from that which relies on a central assessment and a retrospective reporting paradigm. There is little evidence that a geographically distant and temporally isolated analysis is an effective impetus to drive quality and positive change. The advantage of this risk-adjusted chart analysis is that an ICU such as the RBH ICU can continuously monitor performance locally. Although we demonstrated neither a lasting deterioration nor an improvement, analysis did recognise variations from a benchmark performance level. Where prospective monitoring of risk-adjusted mortality shows a persistent and real change, management and clinicians are well placed to respond rapidly with suitable investigation and corrective strategies if necessary. Delays in recognition are imposed by the delays inherent in a system of central collation and may cause a clinical opportunity for recognition to be lost. The use of techniques such as the E-O chart and the risk-adjusted *p *chart can minimise delays between data collection and formative analysis.

Where the risk adjustment model consistently underestimates or overestimates the risk of death, it can be difficult to make any assumptions about changes over time. It is desirable (where there are adequate patient data) to locally validate or recalibrate the estimates of risk of death. Using a simple logistic regression model, with the observed outcome as the independent variable and the UK APACHE II estimate as the dependent variable, we recalibrated the UK APACHE II model for RBH. After we plotted the charts again, there was no evidence of change in risk-adjusted outcome at RBH ICU over the period of analysis.

The E-O chart provides a simple, continuously updateable, qualitative display of the effects on risk-adjusted mortality of the whole health care process surrounding intensive care admissions. However, care must be taken not to overinterpret the E-O chart because fluctuations can represent random variations, or real but transient and reversible changes in the quality of care. In either case, tampering could produce more undesirable effects within the system. However, a persistent change in the slope of the E-O chart should prompt a statistical evaluation of the significance of impressions gained. The response time can be improved if 30-day survival is used instead of in-hospital survival [2].

Where a deficiency has been recognised and corrected or an initiative has improved patient outcomes, contemporaneous monitoring would be able to provide additional evidence for the effectiveness of the corrective strategy.

The E-O chart and the risk-adjusted *p *chart are presented in preference to the more technically demanding formal sequential tests such as adaptations of the CUSUM [7,32,38], other sequential probability ratio tests [10] and the Sets method [11], which have also been proposed for analysis of risk-adjusted data in a medical context. These sequential methods are more sensitive to changes in patient outcome [6]. However, we perceive a barrier to their local adoption by hospitals because of the complexity of analysis, unfamiliarity among clinicians and managers and difficulty in translating to clinical practice. The E-O chart offers a rapid and qualitative plot. The risk-adjusted *p *chart offers an easy formal statistical test, comparing the observed and the predicted mortality rate for each sample period.

We present a technique for real-time risk-adjusted analysis that has proved useful in the analysis of local performance in a large district hospital ICU. We have presented this as a practical response to the need to adopt a local responsibility for our unit's process. This is in contrast to, but complements, a centralised surveillance strategy. We have used the data collected for central analysis, and analysed it in a way that provided local formative ICU assessment of mortality rate performance. This approach poses little additional burden in cost and infrastructure.

## Conclusion

We present a simple risk-adjusted approach to outcome monitoring to allow the rapid detection of unplanned systematic changes affecting patient outcomes. We also offer this as a method of tracking the effect of a deliberately imposed change on patient survival, such as may be imposed by changing staff pattern, resources, or the deliberate application of therapy advocated by randomised trials from elsewhere. This complements a centralised national audit and reporting system that provides valuable archival and comparative data but not the contemporaneous analysis necessary for timely formative use. We monitor the global quality of the service with respect to hospital survival offered by this regional ICU, benchmarking against national UK standards.

## Key messages

• 	Health care is a complex adaptive system. Any change in such a clinical environment will have both predictable and unpredictable effects.

• 	Patient survival from intensive care is influenced by both clinical and organisational factors.

• 	There should be a greater emphasis on continuously monitoring the effect of an entire clinical environment on patient survival, rather than just isolated pockets of applied evidence.

• 	The cumulative risk adjusted mortality chart and the risk adjusted *p *chart are simple techniques to provide near real-time monitoring of the effect of the whole process on survival of patients in intensive care.

• 	This real-time monitoring supplements rather than competes with larger centralised databases, which provide powerful retrospective comparative audit and archival data.

## Abbreviations

APACHE II = Acute Physiology and Chronic Health Evaluation version II; CMP = Case Mix Programme; CUSUM = cumulative sum; E-O = expected minus observed; ICNARC = Intensive Care National Audit & Research Centre; ICU = intensive care unit; RBH = Royal Berkshire Hospital; SMR = standardised mortality ratio.

## Competing interests

The authors declare that they have no competing interests.

## Authors' contributions

JC was responsible for the conception of the study, data acquisition and verification and drafting the manuscript. DC preformed the statistical analysis and was responsible for the conception of the study and the drafting of the manuscript. RI was responsible for the conception of the study and for data acquisition and verification of the data. All authors read and approved the final manuscript.

## Supplementary Material

Additional File 1 A Microsoft Word file containing a description of the construction of risk adjusted control charts. Click here for file

## Figures and Tables

**Table 1:** Comparison of patients excluded from scoring between patients in CMP UK database and those admitted to the RBH

Parameter	CMP database, Jan to Dec 2003		RBH, Jan 2003 to Jun 2004	
	Number	%	Number	%
Total number of admissions	55,936	100.00	731	100.00
Age at admission <16 years	978	1.75	17	2.33
Length of stay in unit <8 hours	3,636	6.50	39	5.34
Admission for primary burns	121	0.22	0	0.00
Admission after coronary artery bypass grafting	36	0.06	0	0.00
Readmission within the same hospital stay	2,911	5.20	34	4.65
Transferred in from another critical care unit	1,836	3.28	27	3.69
Missing all 12 physiological variables	1,488	2.66	26	3.56
Total number included for scoring	44,930	80.32	588	80.44

CMP, Case Mix Programme; RBH, Royal Berkshire Hospital.

**Table 2:** Comparison between CMP UK database and admissions to RBH ICU

Parameter	CMP database, Jan to Dec 2003		RBH, Jan 2003 to Jun 2004	
	*n*	Value	*n*	Value
Case mix				
Age, years (mean ± SD)	55,936	60 ± 19.3	731	57 ± 20.4
Median age, years	55,936	64	731	62
Gender male, %	32,035	57.3	731	54.7
APACHE II				
APACHE II score (mean ± SD)	45,099	16.7 ± 7.4	589	20.8 ± 8.3
UK mortality probability, % (mean ± SD)	45,099	26.3 ± 22.2	589	33.5 ± 26.1
Median UK mortality probability	45,099	19.1	589	25.8
Surgical status, %	55,936	100	731	100
Nonsurgical, %	30,545	54.6	450	61.6
Elective/scheduled surgery, %	13,825	24.7	168	23
Emergency/urgent surgery, %	11,494	20.5	113	15.5
'Surgery, unknown classification', %	72	0.1	0	0
Outcome				
Mortality, all patients, %	45,099	27.7	731	28.9
Mortality, included for scoring^a^, %	53,464	29.3	589	26.0
Standardised mortality ratio		1.11		0.78
Activity				
Median length of stay (days)	55,936	2.11	731	1.8

^a ^in accordance with the ICNARC Casemix programme

APACHE, Acute Physiology and Chronic Health Evaluation; CMP, Case Mix Programme; ICU, intensive care unit; RBH, Royal Berkshire Hospital.
