# Supplementary material for: Process monitoring in intensive care with the use of cumulative expected minus observed mortality and risk-adjusted p charts
Source: Crit Care. 2006 Feb 14;10(1):R28. doi: 10.1186/cc3996 (PMC1550849; doi:10.1186/cc3996)
Supplement: Additional File 1 — A Microsoft Word file containing a description of the construction of risk adjusted control charts. [file cc3996-S1.doc]

## Appendix 1

## Construction of Risk Adjusted Control Charts.

The following notation and formulae are used for the charts.

$Y_{ij}$ is the outcome for patient *j* in sample *i .* If the patient dies, $Y_{ij}=1$ and if the patient survives, $Y_{ij}=0$*.* The estimate of the probability of patient death provided by the risk adjustment tool is $\hat{\pi}_{ij}$ so the estimate of the probability of survival is $1-\hat{\pi}_{ij}$

- E – O Chart

For a series of patient series, the value for the cumulative E – O statistic *C* at patient *n* is

- Risk Adjusted *p* Chart

The expectation of the outcome of each patient $\hat{E}\left(Y_{ij}\right)$ is estimated by the risk adjustment model:

The variance of that estimate is approximately

The observed mortality rate $R_{i}$ for sample i is

$$R_{i}=\frac{\sum Y_{ij}}{n_{i}}$$

and the predicted or expected mortality rate, $\hat{E}\left(R_{i}\right)$ is

The variance of the observed mortality rate,

The RA *p* chart compares $R_{i}$ to $\hat{E}\left(R_{i}\right)$with control limits calculated around $\hat{E}\left(R_{i}\right)$. The control limits are defined as multiples of the standard deviation (the square root of the variance). Let a be the number of standard deviations, then

- Chart Design and Parameter Choice.

Under plausible clinical scenarios, the width of the control limits, the number of cases and the casemix of the population under analysis are the important determinants of the sensitivity and specificity of the risk adjusted p chart. These are common considerations for design and parameter choice for any of the risk adjusted control charts. Although the ICU population represents a diverse range of diagnosis, death in ICU is relatively common and occurs in roughly 10 - 35% of patients. These charts are robust and more suitable under these conditions, than for monitoring for rate events, such as in cardiac surgery (mortality rate: 1 – 5%) and population surveillance, in the “Shipman” detection scenario.

The control limits are specified at 2 standard deviations above and below the expected mortality rate. The average number of cases admitted before a false alarm (false positive) and before a true signal (true positive) can be examined by the calculating the average run length to signal across a plausible range of clinical scenarios of better or worse performance. Performance of the charts can be examined by using approximations of distribution, by simulation or by exact methods. An acceptable chart design can be chosen, and the effect of repeated analysis can be accounted for.

It is convenient to consider clinical scenarios as changes in risk of death in terms of an altered odds ratio (*OR*) where it is the odds of death rather than the absolute risk of death that changes.

The performance of a control chart is assessed in terms of average run length in-control (OR = 1) where the model predictions match the observed outcomes. Similarly where the system is out-of-control (OR ≠1) the model predictions are either above or below the observed mortality rate.

A computer simulation was used to determine the way a chart would perform, and whether it would be clinically useful. To do this, patients are chosen at random with replacement from a relevant case mix in blocks. To design a chart for RBH ICU, the historical casemix was used, and the consequences of patient blocks of 10, 20, 30, 50, 70 and 100 cases were examined. Simulated outcomes were allocated as a series of Bernoulli trials, based on specified OR and the out-of-control risk of death $\pi_{ij}^{A}$. For Figure A1, the average run length is expressed in average number of cases before an “observed” mortality rate of a sample falls outside the control limits. Five thousand simulations were performed at each value for OR in the range 0.5 – 2, in increments of 0.25.

The average run length of each block size with 2 standard deviation control limits is approximately 20 times the block length when the model is accurately predicting risk of patient death, ie *OR* = 1. Thus the larger the size of the samples, the more patients you will admit before a false alarm. However, the larger block sizes also impose a delay before detection of altered odds of death, where the risk adjusted mortality performance has risen or fallen compared to the predicted mortality. For a block size of 30 patients, the false alarms will occur on average of 897 patients or every 2 years at the RBH. However, an increase of odds of death of 50% will evoke a signal after 183 patients or by the 6th month on average. For samples of 10 patients, false alarms occur approximately annually, though 50% increase in odds of death will be not be detected much more rapidly at 179 patients. In contrast blocks of 100 patients will signal falsely on average every 5 years (2184 patients), but will detect a 50% increase in odds of death on average at about 9 months (261) patients.

As a compromise between false alarm rate, and ability to detect true differences in mortality rate, block size of 30 patients was chosen.

# Figure A1: Average Run Length for Risk Adjusted p Chart

5000 Simulations performed on Cases from Royal Berkshire Hospital Intensive Care Unit

# Sample sizes of 10, 20, 30, 50 70 and 100 patients, Control limits set at +/- 2 standard deviation
